# Insight into the Mode of Action of Celangulin V on the Transmembrane Potential of Midgut Cells in Lepidopteran Larvae

**DOI:** 10.3390/toxins9120393

**Published:** 2017-12-06

**Authors:** Yingying Wang, Jiwen Zhang, Mingxing Feng, Wenjun Wu, Zhaonong Hu

**Affiliations:** 1 College of Plant Protection, Northwest A&F University, Yangling 712100, China; wang553135575@163.com (Y.W.); fengmx2010@126.com (M.F.); 2 Provincial Key Laboratory for Botanical Pesticide R&D of Shaanxi, Yangling 712100, China; zhangjiwen@nwsuaf.edu.cn (J.Z.); wuwenjun@nwsuaf.edu.cn (W.W.); 3 Shandong Institute for Product Quality Inspection, Jinan 250102, China

**Keywords:** *Celastrus angulatus*, Celangulin V, *Mythimna separata*, Agrotis ipsilon, midgut apical membrane, transmembrane potential, V-ATPase

## Abstract

Celangulin V (CV) is the main insecticidal constituent of *Celastrus angulatus*. The V-ATPase H subunit of the midgut cells of lepidopteran larvae is the putative target protein of CV. Here, we compared the effects of CV on the midgut membrane potentials of *Mythimna separata* and *Agrotis ipsilon* larvae with those of the Cry1Ab toxin from *Bacillus thuringiensis* and with those of inactive CV-MIA, a synthetic derivative of CV. We investigated the changes in the apical membrane potentials (*V_am_*) and basolateral membrane potentials (*V_bm_*) of the midguts of sixth-instar larvae force-fed with the test toxins. We also measured the *V_am_* and *V_bm_* of larval midguts that were directly incubated with the test toxins. Similar to the effect of Cry1Ab, the *V_am_* of CV-treated midguts rapidly decayed over time in a dose-dependent manner. By contrast, CV-MIA did not influence *V_am_*. Meanwhile, the *V_am_* of *A. ipsilon* larval midguts directly incubated with CV decayed less than that of *M. separata* larval midguts, whereas that of larvae force-fed with CV did not significantly change. Similar to Cry1Ab, CV did not affect the *V_bm_* of isolated midguts. CV significantly inhibited V-ATPase activity in a dose-dependent manner. Therefore, CV initially inhibits V-ATPase in the apical membrane and affects intracellular pH, homeostasis, and nutrient transport mechanisms in lepidopteran midgut cells.

## 1. Introduction

Insecticides are key control agents of insect pests that threaten agricultural production and spread diseases [1]. However, the long-term and extensive use of insecticides has resulted in a series of serious negative impacts, such as pesticide resistance, lethal effects on non-target organisms, and environmental contamination. Given that an increasing number of insecticides have been subjected to regulatory restrictions or banned [2], biologically based alternatives to chemical insecticides have been extensively explored [3]. The use of plant-derived secondary metabolites as insecticides has also been investigated because of their superior effectiveness, safety, and ecological acceptability [4]. Active insecticide components such as pyrethrin, nicotine, and rotenone have been successfully isolated from numerous plants and widely utilized in pest management [5,6,7,8,9]. With significant advancements in natural product science, such compounds have been used or characterized as primary compounds in synthetic chemistry or as probes for new and effective biochemical targets in pesticide research and development [10].

The medicinal and pesticidal characteristics of Chinese bittersweet (*Celastrus angulatus* Maxim) are well known and have been reviewed earlier [11,12]. Its root bark extract has been registered and commercialized as an insecticide in China [13]. It also contains numerous chemical substances, such as Celangulin V (CV), a major insecticidal component [14,15]. CV exerts an extreme insecticidal effect on *Mythimna separata* larvae and causes various symptoms, such as excitation, tremors, and body fluid loss [11]. Meanwhile, intoxicated insects suffering from body fluid loss exhibit time-dependent melanization [16]. Similar to insect death caused by the *Bacillus thuringiensis* (*Bt*) toxin, insect death caused by CV occurs through midgut disturbance [11]. Wu et al. [17] proposed that similar to the *Bt* toxin, CV is an insect digestive agent that acts on midgut membranes. Meanwhile, immuno-electron microscopy revealed that CV is associated with the insect midgut epithelium [18]. Histopathological changes have demonstrated that CV ingestion can severely damage the midgut cells of *M. separata* larvae [19]. Lu et al. [20,21] speculated that the V-ATPase of the midgut cells of *M. separata* larvae is a putative target of CV. Through affinity chromatography and liquid chromatography quadrupole time-of-flight mass spectrometry, they validated that the V-ATPase H subunit is the target binding site of CV. However, the insecticidal mechanism of CV remains elusive.

Two lepidopteran insect species, *M. separata* and *Agrotis ipsilon*, are used as test insects in *C. angulatus* research because of the selective larval toxicity of *C. angulatus*. In particular, *A. ipsilon* is unsusceptible to CV, whereas *M. separata* is sensitive [11]. In the present study, as previously described [22,23], we used the intracellular microelectrode recording technique to compare the effects of CV on the apical potentials (*V_am_*) and basolateral membrane potentials (*V_bm_*) of the larval midgut with those of Cry1Ab from *B. thuringiensis* and inactive CV-MIA, a synthetic derivative of CV. We found that the *V_am_* of *M. separata* is significantly attenuated regardless of whether CV was force-fed to larvae or directly incubated with isolated midguts. These results further implied that CV acts on V-ATPase by disturbing transepithelial transport function. Our findings not only validated our hypothesis, but also provided a basis for elucidating the insecticidal molecular mechanism of CV and its analogs.

## 2. Results

### 2.1. Effects of CV Ingestion on Midgut Transmembrane Potentials

**Figure 1:**
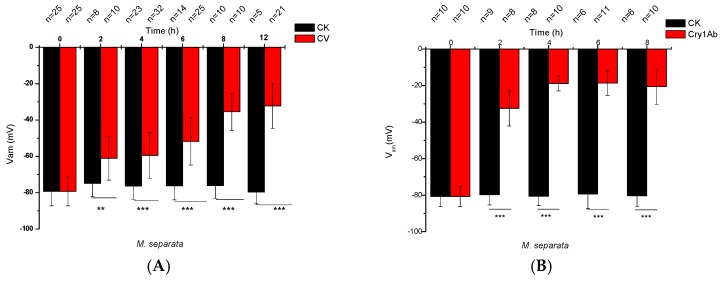

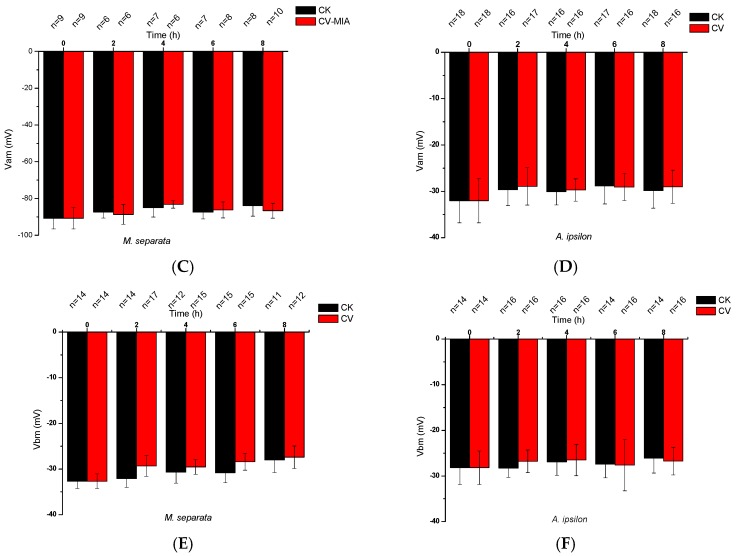
Effects of Celangulin V (CV) ingestion on the midgut *V_am_* and *V_bm_* of sixth-instar *M. separata* and *A. ipsilon* larvae. *M. separata* and *A. ipsilon* larvae were force-fed with 25 μg of CV/CV-MIA (CV-MIA is a synthetic derivative of CV) dissolved in 1 μL dimethyl sulfoxide (DMSO) or with 1 μg of Cry1Ab dissolved in 1 μL 32 K solution. Then, changes in *V_am_* and *V_bm_* over time were measured. Panels (**A**–**D**) show the results for *V_am_*; whereas panels (**E**,**F**) display those for *V_bm_*. CK indicates data for control larvae, which were force-fed with 1 μL DMSO or 32 K solution. The *V_am_* or *V_bm_* is the average value of the measured membrane potentials that remained stable for longer than 5 min. The presented data are expressed as mean ± standard deviation (SD) for 7 to 28 independent larvae. Statistical significance was determined through Student’s *t*-test, and significant values were set at ** *p* ≤ 0.01 and *** *p* ≤ 0.001.

*M. separata* and *A. ipsilon* larvae were force-fed with 25 μg of CV dissolved in 1 μL of dimethyl sulfoxide (DMSO). The *V_am_* and *V_bm_* of midgut cells over time were then directly measured (Figure 1). Figure 1A presents the effects of CV ingestion on *V_am_*. The initial *V_am_* of the DMSO-treated control was −79.30 ± 7.96 mV (*n* = 25) and remained stable for 12 h (Figure 1A). By contrast, after CV ingestion, *V_am_* rapidly depolarized with time. *V_am_* decayed to −61.02 ± 12.07 (*n* = 8) after 2 h, −59.47 ± 12.54 (*n* = 32) after 4 h, −51.78 ± 13.02 (*n* = 25) after 6 h, −35.47 ± 10.23 (*n* = 10) after 8 h, and −32.18 ± 12.44 mV (*n* = 21) after 12 h.

The effects of activated Cry1Ab on the *V_am_* of *M. separata* are similar to those of CV. The initial *V_am_* of the control larvae, which only ingested 32 K solution, was −80.71 ± 5.58 mV (*n* = 10) and remained stable for longer than 8 h (Figure 1B). However, the *V_am_* of larvae that ingested Cry1Ab directly depolarized to −32.42 ± 9.69 (*n* = 8), −18.80 ± 4.12 (*n* = 10), −18.61 ± 6.71 (*n* = 11), and −20.59 ± 9.69 mV (*n* = 10) at 2, 4, 6, and 8 h, respectively. By contrast, CV-MIA, which exerts no insecticidal activity on *M. separata* larvae, did not affect *V_am_* (Figure 1C), and the *V_am_* of *A. ipsilon* larvae that ingested CV did not significantly change throughout 8 h of recording (Figure 1D). The *V_bm_* values of both larval species are shown in Figure 1E,F. The initial *V_bm_* values of the control *M. separata* and were *A. ipsilon* larvae −32.66 ± 1.60 (*n* = 14) and −28.19 ± 3.66 mV (*n* = 14), respectively. Correspondingly, compared with the control treatment, CV did not affect the *V_bm_* of both larval species. Similarly, CV-MIA and Cry1Ab had no effect on *V_bm_* (data not shown).

These results suggested that CV is highly active in the apical membranes of *M. separata* larvae but is inactive in those of *A. ipsilon*. Moreover, CV does not affect the basolateral side of midgut cells given that CV treatment did not significantly influence the *V_bm_* of both insect species. Evidently, CV acts specifically on the apical membranes of the midgut cells of *M. separata* larvae.

Table 1 shows the midgut transepithelial voltages (*V_t_* = *V_bm_ − V_am_*) of *M. separata* larvae under different treatments. Treatment with CV or Cry1Ab gradually decreased midgut *V_t_* over time until it finally approached zero. The control treatment, which contained DMSO, 32 K, and inactive CV-MIA, did not significantly influence *V_t_*. This result further demonstrated that the insecticidal action of CV is correlated with changes in *V_t_*.

### 2.2. Direct Effects of CV on Midgut Transmembrane Potentials

**Figure 2:**
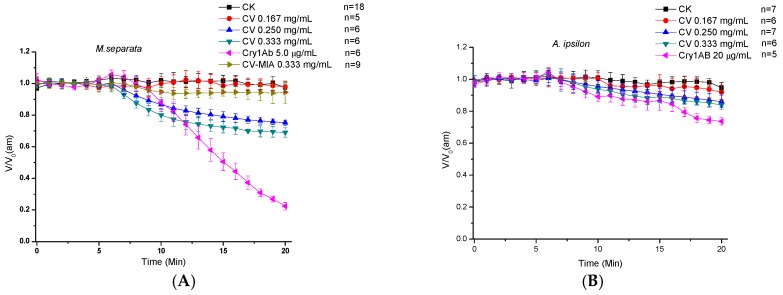

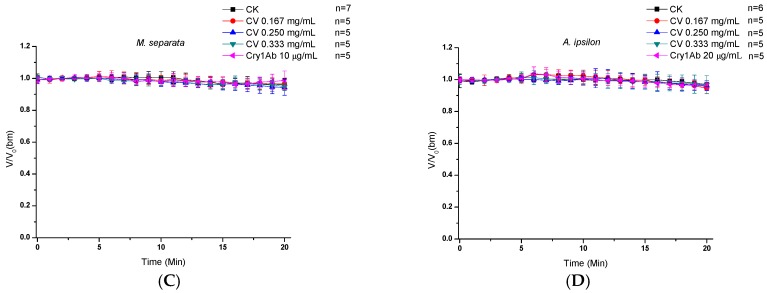
Direct effects of CV on the midgut *V_am_* and *V_bm_* of sixth-instar *M. separata* and *A. ipsilon* larvae. When membrane potential remained stable for longer than 5 min, an equal volume of recording bath was directly replaced with 32 K solution containing the test toxins. The concentrations of CV in the recording bath were 0, 0.167, 0.260, and 0.333 mg/mL, whereas the CV-MIA was 0.333 mg/mL. Activated Cry1Ab was adopted as the positive control, and its concentration in the recording bath was set at 5.0 or 10 μg/mL to *V_am_* and *V_bm_* measurement for *M. separata*, respectively; the concentrations was 20 μg/mL to *V_am_* and *V_bm_* measurement for *A. ipsilon*. Panels (**A**,**B**) show the results of *V_am_* measurement; whereas Panels (**C**,**D**) show those of *V_bm_* measurements. *V* represents *V_am_* or *V_bm_*, which was measured with time, and *V*_0_ is the average values of membrane potential measured during stabilization before toxin addition. The data are presented as mean ± SD for 5 to 18 independent larvae.

When the midgut membrane potentials remained stable for longer than 5 min, the direct effects of CV on the *V_am_* and *V_bm_* were investigated by replacing the recording bath with an equal volume of 32 K solution containing the test toxins. Figure 2 shows the influences of different CV concentrations on the *V_am_* and *V_bm_* of both larval species. At the low concentration of 0.167 mg/mL, CV negligibly affected *V_am_*. However, depolarization occurred in a concentration-dependent manner (Figure 2A). The depolarization rates of 0.250 and 0.333 mg/mL CV reached 19.7% ± 3.1% and 26.8% ± 5.2%, respectively, after 10 min and 24.2% ± 1.7% and 30.6% ± 6.3%, respectively, after 15 min. Although CV can influence the apical membrane of *A. ipsilon* larvae, the decline in the potential difference of the midguts of *A. ipsilon* larvae was less than that in the midguts of *M. separata* larvae. Even at the high concentration of 0.333 mg/L, the depolarization rate of CV was only 14.8% ± 9.4% and 19.3% ± 8.2% after 10 and 15 min, respectively (Figure 2B). Moreover, CV only slightly affected the *V_bm_* of both larvae, even at the high concentration of 0.333 mg/mL (Figure 2C,D).

The recording bath solution was replaced with an equal volume of 32 K solution containing activated Cry1Ab as the positive control. The direct effects of Cry1Ab on *V_am_* and *V_bm_* were similar to those of CV ingestion. At the concentration of 5 μg/mL, the activated Cry1Ab significantly affected *V_am_* of *M. separata* larvae (Figure 2A). The depolarization rate of Cry1Ab was higher than that of CV. Cry1Ab reduced membrane potential by 50% within 10 min. However, CV, has a faster response time than Cry1Ab. Moreover, 10 μg/mL of Cry1Ab did not influence *V_bm_* (Figure 2C). The results of CV-MIA incubation were similar to those of CV-MIA ingestion, and *V_am_* was unaffected by CV-MIA at a high concentration of 0.333 mg/mL (Figure 2A).

### 2.3. Effects of CV on V-ATPase Activity in Midguts of M. Separata Larvae

**Figure 3:**
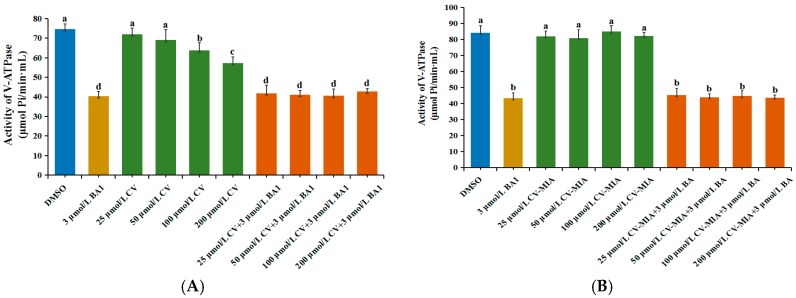
Effects of CV and CV-MIA on V-ATPase in the midgut epithelial cells of *M. separata* larvae. The data are presented as mean ± SD. Different lower-case letters indicate significant differences between treatments by ANOVA analysis (*p* < 0.05). All experiments in this study were repeated more than three times.

The influences of CV and CV-MIA on the midgut V-ATPase of *M. separata* larvae are presented in Figure 3. V-ATPase activity was significantly inhibited by treatment with 200 and 100 µmol/L of CV, with inhibition ratios of 23.41% and 11.80%, respectively (Figure 3A). The positive control bafilomycin A1 (BA1)—a well-known V-ATPase inhibitor—exerted a significant suppression effect and achieved an inhibition ratio of up to 46.05%. To thoroughly understand the effect of CV on V-ATPase, larvae were simultaneously treated with CV and BA1. V-ATPase activity was significantly inhibited under all four combined treatments with different CV concentrations, but its response did not significantly differ from that under treatment with the positive control BA1. The effects of different CV-MIA concentrations on V-ATPase were not significantly different from those of the DMSO control. These results further indicated that CV is correlated with midgut V-ATPase.

## 3. Discussion

The above results demonstrated that CV depolarizes the apical membranes of the midgut cells of *M. separata* and *A. ipsilon* larvae in a concentration- and time-dependent manner. Furthermore, the effect of CV on the midgut cell membrane is similar to that of Cry1Ab. Notably, however, the effect of CV on the apical membrane is more potent than that on the basolateral membrane. Enzymology studies indicated that CV significantly inhibits V-ATPase activity in the midguts of *M. separata* larvae. These results suggested that CV may exert a relatively specific effect on the apical membrane.

Our results for Cry1Ab-treated *M. separata* larvae are consistent with those of previous reports, [22,23,24] which indicated that Cry1Ab only significantly influences the apical membranes of lepidopteran larvae. In general, activated Cry1Ab toxins initially bind to specific receptors, including aminopeptidase N (APN) or alkaline phosphatase, insert into apical membranes, and then form pores in the midgut lumen [25,26]. These pores abolish ionic gradients and thus induce midgut lysis, which eventually leads to insect death [27,28,29,30]. The depolarization rates of the *V_am_* of *M. separata* larvae reached 50% after 10 min of incubation with 5 μg/mL of Cry1Ab. CV is a small molecular compound with a *β*-dihydroagarofuran skeleton. Theoretically, in contrast to the *Bt* toxin, it cannot form pores in the apical membrane. We found that although the effects of CV on the *V_am_* of *M. separata* are less intense than those of Cry1Ab, the action on *V_am_* of the former is faster than that of the latter. Although a previous report showed that the V-ATPase of the insect midgut is a possible target of *Bt* toxins [31], we maintain that the influence of Cry1Ab on *V_am_* is mainly due to pore formation in the apical membrane [32]. The action mechanisms of Cry1Ab promote *V_am_* depolarization more effectively than those of CV, which may only target V-ATPase. The similar changes in *V_am_* after force feeding CV and *Bt* toxin also illustrate this point.

The couplet system of V-ATPase and the K^+^/H^+^ antiporter—which is located in the apical membrane of the midgut goblet cells of lepidopteran larvae—is the basis of transmembrane potential difference, intracellular ion gradient, and nutrition absorption/transport [33,34]. V-ATPase in the midgut cells of *M. separata* larvae was previously speculated to be a putative target of CV [20]. The V-ATPase H subunit has been validated as the target binding protein of CV [21]. This view was confirmed by systemic enzymology studies. Early results showed that CV does not influence APN activity [21]. Here, we showed that V-ATPase is significantly inhibited by CV but is unaffected by inactive CV-MIA. Our results further suggested that the midgut V-ATPase of *M. separata* is a target enzyme of CV. Moreover, we speculate that CV can directly target the apical membrane of midgut goblet cells and cause *V_am_* decay. Therefore, V-ATPase may be the most important target of CV, which disturbs normal insect function by disrupting ionic/chemical homeostasis and nutrition absorption/transport throughout the whole midgut via a mechanism that involves goblet and columnar cells. Ultimately, these effects cause irreversible midgut damage.

*V_am_* is significantly depolarized under CV ingestion or CV incubation relative to that under treatment with inactive CV-MIA. However, high concentrations of CV-MIA do not affect *V_am_*. These responses are consistent with corresponding bioassay results and further proved that the insecticidal CV only initially targets the midgut apical membranes and subsequently affects midgut ionic/chemical homeostasis, which results in an abnormal *V_am_*. In insects, disorders in midgut ionic/chemical homeostasis causes diarrhea and vomiting, body water and hemolymph loss, midgut rupture, and death.

Consistent with larval bioassay toxicities [11], the depolarizing effect of CV on the apical membranes of non-susceptible *A. ipsilon* is less intense than that on the apical membranes of susceptible *M. separata.* The significant difference in *V_am_* between the two larvae may be ascribed to different apical membranes due to dissimilar living habits and physiological or biochemical characteristics; these differences ultimately resulted in their divergent sensitivity to exogenous substrates [22]. However, the specific reasons for the different sensitivities of the two insect species remain to be further investigated.

On the basis of the above results, we concluded that CV may target V-ATPase in the apical membrane of the midgut goblet cells of *M. separata*. CV may initially trigger the collapse of the electrochemical gradient of midgut apical membranes. This effect subsequently causes the loss of normal physiological function. Meanwhile, the specific mechanism of this effect remains unclear and requires further research.

## 4. Materials and Methods

### 4.1. Insects

As described previously [22], laboratory colonies of *M. separata* and *A. ipsilon* were maintained at 25 °C, relative humidity of 75–80%, and photoperiod of 16 h:8 h (light:dark) to obtain uniform and healthy larvae for experiments.

### 4.2. Solutions

The buffer solution used for the determination of midgut membrane potentials was the same as that previously reported [22], and consisted of 32 mmol/L KCl, 5 mmol/L CaCl_2_, 5 mmol/L MgCl_2_, 166 mmol/L sucrose, and 5 mmol/L Tris–HCl (pH = 8.0).

### 4.3. Test Toxins and Chemicals

Trypsin-activated purified Cry1Ab was purchased from Envirologix (Portland, ME, USA). The toxin was dissolved in 25 mmol/L Tris–HCl (pH = 9.4) and used to prepare stock solutions containing 2 mg/mL Cry1Ab. The stock solutions were stored at 4 °C.

**Figure 4:**
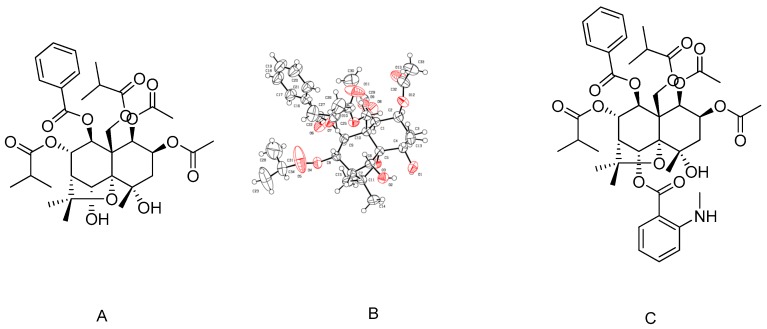
(**A**) Structure of CV; (**B**) X-ray crystal structure of CV; and (**C**) structure of CV-MIA.

Technical-grade (purity > 95%) CV and CV-MIA (a synthetic derivative of CV) were prepared by the Institute of Pesticide Science (Northwest A&F University, Yanglin, China). The molecular formula of CV is 2β,8α-diacetoxy-9β-benzoyloxy-1β, 12-diisobutanoyloxy-4α,6α-dihydroxy-β-dihydroagarofuran. The structures of CV and CV-MIA are shown in Figure 4. Bafilomycin A1 (BA1) was purchased from Sigma-Aldrich (Shanghai, China).

CV, CV-MIA, and BA1 were dissolved in DMSO and diluted in corresponding buffer solutions upon experimentation.

### 4.4. Treatment

Sixth-instar larvae were starved for 8 h, and each larva was force-fed with 1 μL of 25 μg/μL test compound or 1 μL of 1 μg/μL activated Cry1Ab [35]. The same batch of larvae fed with DMSO or 32 K solution was set as the control group. The treated larvae were reared in 24-well cell culture plates and fed normally for membrane potential measurements after 0, 2, 4, 6, 8, and 12 h.

To determine the immediate effects of the test chemicals on larval midguts, stock solutions were diluted with recording buffer solution. The final DMSO concentration in 32 K solution was less than 0.1%.

### 4.5. Membrane Potential Measurements

Membrane potentials (*V_am_* and *V_bm_*) were measured as previously described [22]. A pair of microdissection scissors was used to transversely excise the midgut from a sixth-instar larva. Both ends of the midgut would curl back onto themselves upon excision. Thus, for *V_am_* measurement, a segment of the midgut was aspirated into a glass pipette from one end until its other end curled around the pipette mouth, exposing only its interior surface (as apical side). To expose the middle section of the midgut as the basolateral side for *V_bm_* measurement, each end of a midgut segment was separately aspirated into a glass pipette. The glass pipettes were then lowered near the bottom of the recording bath, and the midguts were immersed into a 32 K solution bath. Midgut cells were impaled with a glass microelectrode filled with 1 mol/L KCl. A useful electrode provides a tip resistance of 50 and 150 MΩ in 32 K solution. *V_am_* or *V_bm_* signals were amplified, acquired, and analyzed by an electrophysiological setup of Axon Instruments (Foster City, CA, USA), including an Axoclamp 900A, a Digidata 1440A analog-to-digital board, and its pCLAMP 10.2 software. Impalement was considered successful when *V_am_* and *V_bm_* fluctuations did not exceed 0.5 and 0.1 mV/min, respectively. Stable membrane potentials were measured for at least 5 min as the control (*V*_0_), and an equal volume of recording bath was replaced with 32 K solution containing the test toxin. Measured potentials (*V*) were normalized relative to the potentials (*V*_0_) measured immediately before the bath solution was replaced (*V*_0_). All experiments were executed at room temperature. Data were presented as mean ± SD for more than five larval midguts. All statistical analyses were conducted with unpaired Student’s *t*-tests, and differences were considered significant at *p* ≤ 0.05.

### 4.6. V-ATPase Activity Measurements

Midguts isolated from fifth-instar *M. separata* larvae were transferred to fresh Ringer’s solution and immediately frozen in liquid nitrogen as described earlier [21]. The preparation of crude membrane pellet and the measurement of V-ATPase activity were then performed as described by Tiburcy et al. [36]. The protein pellet concentration was determined by Bradford assay. The concentration of bafilomycin A1—a V-ATPase inhibitor used as the positive control—was 3 µmol/L in 160 µL reaction buffer. CV and CV-MIA concentrations were set as 25, 50, 100, and 200 µmol/L. The amount of inorganic phosphate produced in the reaction was determined as described by Wieczorek et al. [37]. Each treatment was performed with at least three biological replicates. Differences between treatments were analyzed by ANOVA analysis (*p* < 0.05).

## Figures and Tables

**Table 1:** Impacts of ingesting CV on the *V_t_* of midgut cells from *M. separata* larvae.

Time	*V_t_*/mV				
	DMSO	CV + DMSO	CV–MIA + DMSO	32 K	Cry1Ab + 32 K
0	56.6	57.1	60.3	57.0	57.0
2	52.6	31.7	58.9	53.2	6.0
4	55.7	30.0	53.5	54.5	0
6	55.4	23.4	57.3	52.1	0
8	58.1	8.1	56.5	50.7	0

Note: *V_t_* (mV) = *V_bm_* − *V_am_*.
